# Structure Characterization and Mechanical Properties of Acidity-Induced Helix of Alginate and Fibers

**DOI:** 10.3390/ma18112619

**Published:** 2025-06-03

**Authors:** Jinhong Yang, Na Sun, Xuelai Xie, Zhangyu Feng, Na Liu, Kai Wang, Min Lin

**Affiliations:** 1 State Key Laboratory of Bio-Fibers and Eco-Textiles, College of Materials Science and Engineering, Qingdao University, Qingdao 266071, China; 19863709329@163.com (J.Y.); xxl15550311426@163.com (X.X.); feng101811@163.com (Z.F.); 17363607490@163.com (N.L.); 2 College of Textiles and Clothing, Qingdao University, Qingdao 266071, China; sunduanna2009@163.com; 3 Institute of Flexible Electronics (IFE), Northwestern Polytechnical University (NPU), Xi’an 710072, China

**Keywords:** alginate fibers, helical structures, high-toughness, sodium alginate

## Abstract

The brittleness of alginate fibers has limited their biological applications. Enhancing fiber toughness without sacrificing fracture tensile strength is challenging. Herein, an acidity-triggered helical conformational change in alginate is demonstrated to improve fiber toughness. During fiber formation by Ca^2+^ crosslinking, HCl triggers 2_1_-helical and antiparallel twofold helical conformational changes in sodium alginate. The helical structures were confirmed using circular dichroism and X-ray diffraction. Rheological analysis revealed that the helical conformation was flexible and could extend fiber elongation from 9.4 ± 0.6 to 15.3 ± 2.2%, while the fracture tensile strength was slightly enhanced by 12.4%, reaching 308 MPa. Thus, toughness was enhanced by 74%, reaching 35.5 ± 2.1 MJ m^−3^, thereby reducing brittleness. The introduction of helical structures required no significant changes to the wet-spinning process and exhibited good processability. The improved elongation and toughness will broaden the biomedical applications of alginate fibers.

## 1. Introduction

The biocompatible and biodegradable features of alginate make it an attractive polymer matrix for a wide range of biological applications [1,2,3,4,5,6,7]. Benefiting from the rich carboxylate groups in its chemical structure, alginate can easily form gels in the presence of divalent and trivalent cations [8]. Thus, fibers, hydrogels, sacs, and membranes are widely studied by crosslinking alginate with Ca^2+^, Zn^2+^, Cu^2+^, Ba^2+^, etc. [9,10]. Liu et al. described a drinkable alginate liquid, which formed a hydrogel in situ and released the drug into the digestive canal [11]. Kim et al. performed alginate gelation in serum to induce aneurysm embolization and necrosis [12]. Among alginate-based materials, calcium alginate fiber is an important industrial product manufactured through a green wet-spinning process. But due to the extremely fast crosslinking speed, alginate fibers commonly exhibit lots of structural defects like uneven Ca^2+^ distribution, phase separation, and core-sheath dislocation [13,14,15]. Moreover, the high density of the calcium crosslinked structure also hinders the motion of polysaccharide under loading, causing brittle fracture. These defects deteriorate the mechanical properties of alginate fibers and limit their biological applications.

To reduce brittleness, enhancing fiber strain and toughness is an urgent demand. Common methods primarily rely on incorporating compatible toughening agents, such as bacteria cellulose, chitosan, spider silk proteins, and polyethylene glycol, to increase fiber elongation [16]. The intermolecular interactions between alginate and additives could form stronger networks for even stress distribution [17,18,19]. However, plasticizers always result in a lower modulus and reduced fracture tensile strength. For example, the introduction of sodium polyacrylate into alginate networks weakens the mechanical properties of fibers [20].

Inspired by natural protein-based fibers, the helical conformation in the secondary structures can provide sufficient fiber elongation and toughness [20,21,22,23,24,25]. Under stretching, helical structures undergo conformational changes to unfold and provide additional elongation [26]. In spider silk fibers, it is reported that increasing helical conformation from 17.8 to 19.2% could enlarge fiber strain from 12 to 23%, while the fracture tensile strength is not sacrificed [27]. In regenerated silk fibers, silkworm fibers, and wool keratin fibers, an increased content of helical structures can also achieve enhanced fiber elongation [28,29]. Therefore, constructing a helical conformation in alginate fibers may be a feasible approach to reduce brittleness and improve toughness.

**Scheme 1:**
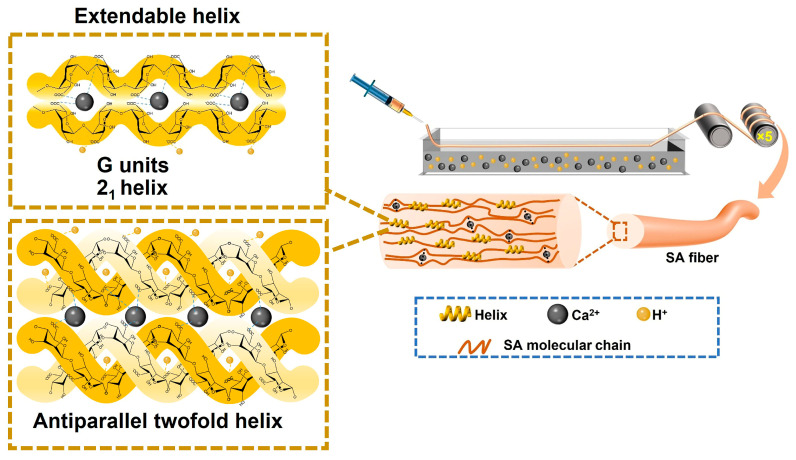
A schematic illustration of alginate fiber production in an HCl/CaCl_2_ coagulation bath. The helix consists of a 2_1_-helical conformation from acidified G units and an antiparallel twofold helix from acidified M units.

The hypothesis of this work is that the elongation and toughness of alginate fibers can be improved by introducing helical structures. This work reports acidity-triggered helical conformational changes in alginate to achieve improved fiber elongation and toughness. Helical conformation mainly consists of 2_1_-helix from G blocks and an antiparallel twofold helix from M blocks (Scheme 1). The helical conformation was shown to have a higher modulus than the random coil, and was extendable under deformation. Consequently, during the wet-pinning process with HCl/CaCl_2_ coagulation, alginate fibers were prepared with increased toughness, which rose from 20.4 to 35.5 MJ m^−3^, and prolonged elongation, extending from 9.4 to 15.3%. The introduction of helical structures required no significant changes to the wet-spinning process and exhibited good processability. The improved toughness will broaden the biomedical applications of alginate fibers.

## 2. Experimental Section

### 2.1. Materials

Sodium alginate (Hyzlin Biology Development Co., Ltd., Qingdao, SA, China) was 273 kDa with M/G = 1.3. HCl (Sinopharm Group, Beijing, China) was analytically pure (36.0～38.0%). Methylene blue (Sinopharm Group, Beijing, MB, China) had ≥70.0% purity. CaCl_2_ (Sinopharm Group, Beijing, MB, China) had a purity of 99.9%. Deionized water (electrical resistivity: 18.25 MΩ) was used in all aqueous solutions.

### 2.2. SA Secondary Structure Analysis

The aqueous SA dispersion (273 kDa, 10^−3^ g mL^−1^) was stored at room temperature under continuous magnetic stirring. The pH of the obtained dispersion was 6.5. For acidified samples, 1 mol L^−1^ HCl was added dropwise to the SA dispersion, and the pH values were separately adjusted to 5, 4, 3, and 2. Then, the samples were tested using circular dichroism (CD) spectral analysis.

Due to the n→π* and π→π* transitions of C=O, pure GGGG blocks have entire negative bands around 200 and 215 nm, and pure MMMM blocks show a characteristic positive band around 190 nm and a negative band around 215 nm. The conformation of GMGM and the irregular sequence is reported to exhibit intermediate CD spectra. These signals were mixed up in the wavelength of 190–250 nm in the CD spectra, which is difficult to observe. To amplify the enantiomeric excess resulting from the helical conformation, methylene blue (MB), a commercial cationic dye, could absorb onto negatively charged alginate and inherit the helix. Thus, the absorption band of MB in 500–700 nm could be used to observe the helical conformation of alginate. In MB stained tests, SA samples were mixed with 1.6 mg mL^−1^ of MB before CD analysis. To eliminate the influence from the absorbed MB, the absorbed amount was evaluated by the absorbance change at 247 nm after filtration and washing. The absorption spectrum of MB exhibited slight changes in different pH values. The changed ratio of absorption intensity at 570 and 665 nm was also determined by the ultraviolet–visible (UV-Vis) absorption spectrum for MBs of different pH.

In X-ray diffraction (XRD) tests, powder SA samples were used. The powders were prepared from 0.5 wt. % SA. For SA treated by HCl, the pH was adjusted to 4. After gently stirring for 1 h, the samples were centrifuged at 3000 r min^−1^ for 10 min and transferred into a vacuum to dry.

### 2.3. Fiber Fabrication Through Wet-Spinning Process

In general, an aqueous SA dispersion (273 kDa, 4 wt. %) was used as the spinning dope, and it was extruded into coagulation baths to produce fibers through injection bump. The spinneret was a metal syringe needle of 0.7 mm in diameter. The value of the shear rate used in producing the fibers was calculated as 148.6 s^−1^. There were two kinds of coagulation baths. The control group was a solution of 5 wt. % CaCl_2_. The acidified group was 5 wt. % CaCl_2_ of different pH values. The pH values were controlled by adding 1 mol L^−1^ HCl. Due to the fast crosslinking rate, instant fiber formation was observed when SA was extruded. By controlling the collecting speed, the stretching ratio was set at 5:1. All fibers were washed with flowing water for 30 s. The pH of the fibers in a wet state was measured by covering pH test papers on fibers. The pH was around 6. When the fibers were soaked in saline at 20 mg mL^−1^ for 24 h, the pH of saline was 6.1. For mechanical property tests, at least 10 fibers from each group were measured in an automated single fiber tester at a strain rate of 20 mm min^−1^. The parameters of the mechanical properties, including breaking tensile strength, breaking strain and linear density (the fiber mass per 1 km, 1 tex = 1 g/km), were automatically obtained. The modulus was calculated from the stress–strain curves in a strain range below 2%. To observe the water absorption of alginate fibers, SA and acidified alginate fibers without drying were soaked in PBS (pH = 7.2) solutions at the concentration of 1 g/20 mL. The weight of fibers and absorbed water were examined at an interval of 24 h.

### 2.4. Characterization Methods

The Fourier transform infrared (FT-IR) spectra of dried SA samples were tested in ATR mode by use of a Nicolet iS50 spectrometer (Thermo Scientific™, Waltham, MA, USA). The data were collected post scanning for 64 cycles in the wavenumber range of 400–4000 cm^−1^.

The water contact angle test was performed on dried films using the optical contact angle tester (Theta, Biolin, Gothenburg, Sweden). The water was dropped onto each sample surface through a syringe at three different positions. The film samples, without Ca^2+^ coagulation, were dried from dispersions of different pH values. All the samples were dried overnight at 60 °C. The recorded contact angle data were from the instant when water droplets dropped on the film.

For circular dichroism (CD, JASCO J-1500, Tokyo, Japan) spectra, the concentration of SA samples was set at 10^−3^ g mL^−1^ to ensure transparency during the measurement. All the CD spectra were tested in a nitrogen atmosphere in the range of 180–250 nm. Data were automatically subtracted from corresponding blank solution samples that contained the same chemicals except for no SA inside. For MB-interacted SA samples, the data were collected in the range of 400–800 nm.

The morphology of alginate fibers was observed by a field emission scanning electron microscope (FEI-SEM, Quanta250FEG, Hillsboro, OR, USA). Before measurement, dried alginate fibers were sprayed with gold in an automatic magnetron ion sputtering instrument (GVC-2000, Gevee, Beijing, China).

The ultraviolet–visible (VU-Vis) spectrophotometer of type T9 was used for absorption spectrum tests. The sample was a mixed dispersion of 10^−4^ g mL^−1^ SA and 1.6 mg mL^−1^ MB, and the wavelength range was set to 200–800 nm.

A gel permeation chromatography (GPC, TDA 305, Malvern, Kassel, Germany) tests used 0.1 M NaNO_3_ as the mobile phase, and the concentration of SA was set at 1 mg mL^−1^.

Thermo-gravimetric analysis (TGA) (TG-290-F3, Netzsch, Selb, Germany) measured the sample in the temperature range of 30–800 °C, and the heating rate was set at 10 °C min^−1^. The retention volume was 16.8 mL for SA of 273 kDa (Figure S1).

The X-ray diffractometry (XRD, D/max-3B, Science Company, Tokyo, Japan) tests of the SA samples were conducted at a voltage of 40 kV and an electric current of 30 mA. The scanning speed was 5° min^−1^ in the range of 2θ = 5–40°. A Cu target was applied (λ = 0.154 nm).

Rheology (DHR-3, TA, New Castle, DE, USA) measurements were performed using a 60 mm sample with a sample thickness of 350 μm. For the viscosity test, the shear rate range at 25 °C was 0.1–1000 s^−1^. The storage modulus (*G*′) and loss modulus (*G*″) based on dynamic frequency were tested at angular frequencies (ω) of 0.1–628 rad s^−1^.

The mechanical properties of SA fiber samples were assessed by an automated single fiber system (AIROBOT, FAVIMAT, Mönchengladbach, Germany). In the tests, fibers were preloaded with a tensile stress of 0.06 cN/dtex (9.6 MPa for alginate fibers). Then, the fibers were stretched at a constant rate of 20 mm min^−1^. Each sample was tested 10 times.

## 3. Results and Discussion

### 3.1. Acidity Regulated Helical Conformation

**Figure 1:**
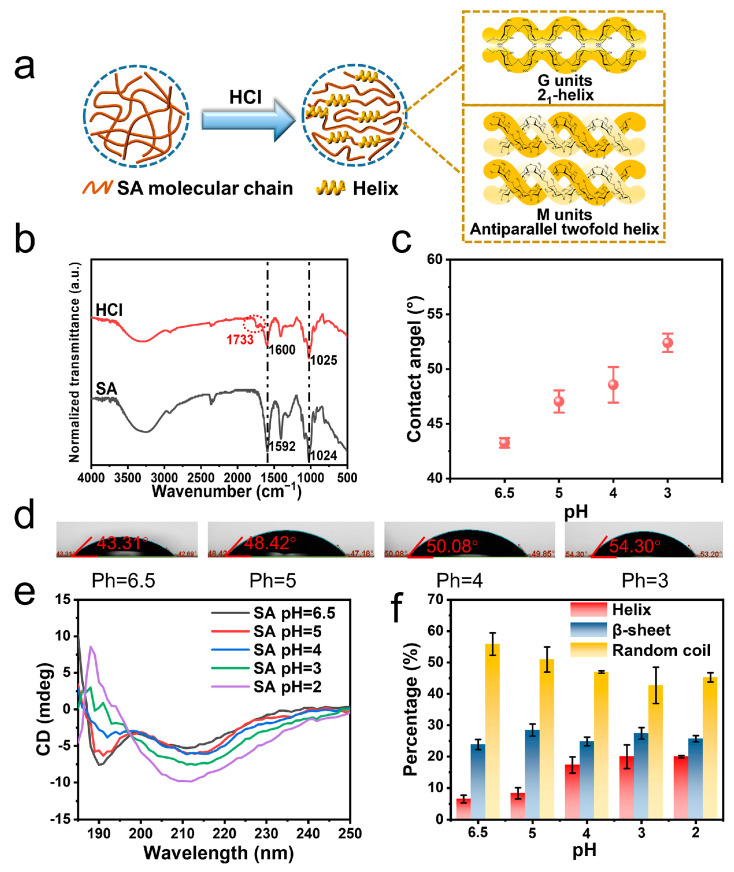
Characterizations of helix in acidified SA. (**a**) Cartoon illustration of HCl-induced helical conformational change of SA. Helix contains 2_1_-helical conformation from acidified G units and antiparallel twofold helix from acidified M units. (**b**) FT-IR spectra of SA and HCl-treated SA. (**c**,**d**) Water contact angle of acidified SA at different pH values (*N* = 3). (**e**) The CD spectrum and (**f**) the content of the secondary structure for acidified SA at different pH values. The molecular weight of SA was 273 kDa.

When an SA of 273 kDa was subjected to acidity, parts of carboxylate groups were converted into carboxylic acid, triggering helical conformational changes (Figure 1a). The protonation generated a peak of C=O stretching vibration at 1733 cm^−1^ next to the ~1600 cm^−1^ peak in the Fourier transform infrared (FT-IR) spectrum, which corresponded to -COOH and -COO^−^, respectively (Figure 1b). The positions of other featuring peaks were not significantly changed compared with SA. The transmittance peak at 1024 cm^−1^ resulted from the stretching vibrations of the C–O groups in the sugar rings. And a weak peak around 2900 cm^−1^ was related to the stretching vibration of C–H groups. Besides this, massive O–H groups and their inner- and inter-molecular hydrogen bonding generated a wide band in the range of 3000–3500 cm^−1^ [30,31]. An increase in water contact angle from 43.3 to 52.4° was observed, while the surface tension decreased from 102.2 ± 2.9 to 75.0 ± 0.9 mN/m (Figure 1c,d). This indicated decreased hydrophilicity for acidified SA. Thus, the secondary structure of SA was changed. It has been reported that the n→π^*^ interaction of carboxyl chromophore could generate characteristic CD signals at 192 and 208–215 nm for SA (Figure 1e and Figure S2) [32,33]. The relative content of helix and β-sheet could be estimated based on these characteristic peaks (Figure 1f, Table S1) [34]. Before HCl treatment, SA was mainly composed of a high content of random coil (55.9%), some β-sheet structures, and a small group of helix (Figure 1f). When the pH was adjusted from 6.5 to 2, the negative absorption band around 210 nm exhibited a stepwise increase in intensity. The content of helix was increased from 6.5% to 20.0%.

**Figure 2:**
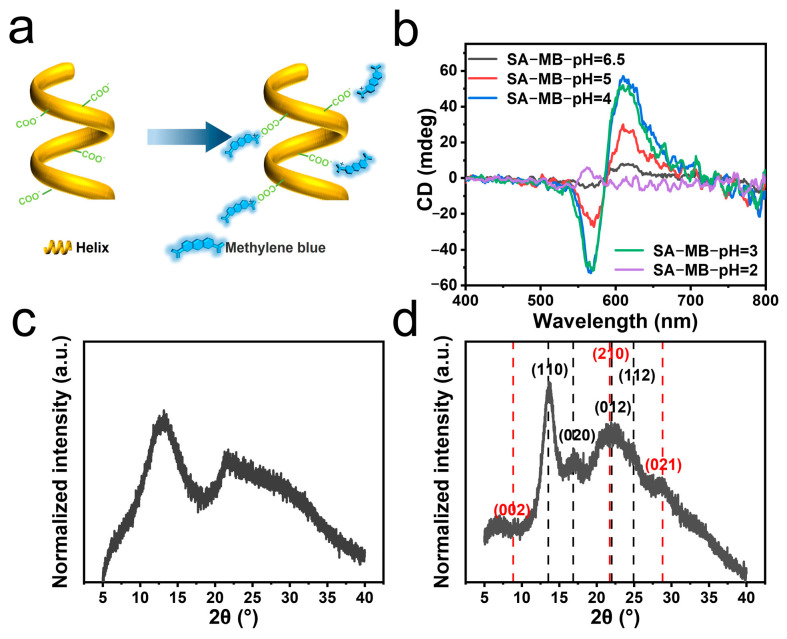
(**a**) Cartoon diagram of SA helix adsorbing MB molecules. (**b**) The CD spectra of the SA/MB mixture at different pH values in the wavelength range of 400–800 nm. The CD signal intensity was calibrated by the absorbed amount of MB for each sample. XRD pattern of SA (**c**) and acidified SA (**d**). The black and red dash lines represent reported crystallographic planes for poly-α-L-guluronic acid and poly-α-L-guluronic acid, respectively [35,36].

To characterize the helix, using dyes to amplify the CD signal is an efficient approach. Considering negative charges on SA, cationic methylene blue (MB) was absorbed onto the helical structures of SA (Figure 2a). Due to geometric symmetry, CD signals of helical structures were amplified by the absorbed MB to the range of 500 to 700 nm (Figure 2b and Figure S3). A negative peak at 570 nm and a positive peak at 665 nm were observed with increased intensity when the pH was decreased from 6.5 to 3, confirming the growing content of helical structures (Table S2). But when the pH was adjusted to 2, the composite of MB and SA precipitated quickly, resulting into undetectable helical signal from the aqueous dispersion. Acidified SA samples were additionally tested by X-ray diffraction (XRD) (Figure 2c,d). This mainly indicates the amorphous structure of SA without HCl treatment. In comparison, improved crystallinity was observed with narrower peaks at 2θ = 13.6 and 16.9° for acidified SA. The featuring peaks at 2θ = 13.6, 16.9 and 22.5° matched well with the (110), (020), and (012) reflections of poly-α-L-guluronic acid, whose cell unit parameters were a = 0.86 nm, b = 1.07 nm and c = 0.87 nm [35]. Hence, the G units in acidified SA were supposed to contain a similar 2_1_-helical conformation with poly-α-L-guluronic acid (Figure 1a). And the absence of (001)reflection in the XRD pattern could also confirm the 2_1_-helical conformation [35]. Meanwhile, the XRD peaks of 2θ = 8.9, 21.7, and 28.8° also matched the (002), (210), and (021) reflections of orthorhombic poly-β-D-mannuronic acid [36]. The unit cell parameters were a = 0.76 nm, b = 1.04 nm, and c = 0.86 nm. Thus, the acidified M units may form the same antiparallel double helical chains, which could further form sheet-like structures with a sheet distance of 0.52 nm. Since naturally extracted alginates are block copolymers, they contain consecutive GGGG blocks, consecutive MMMM blocks, GMGM blocks and random sequences. Before protonation, sodium alginate possessed semi-stiff polymer chains, making it difficult to aggregate or crystalline. After protonation, the GGGG blocks could form a 2_1_-helical conformation and MMMM blocks could form an antiparallel twofold helix. Some irregular helical structures may also exist, but they were not clearly identified here. The reduced electrostatic repulsion and massive level of new hydrogen bonding could help stabilize the changed conformations [26].

### 3.2. Rheological Analysis of Helix

**Figure 3:**
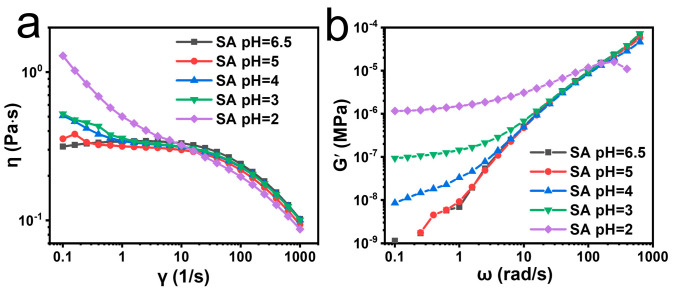
Rheological measurements of acidity-regulated helix in SA dispersions. (**a**) Viscosity (*η*) and (**b**) storage modulus (*G*′) of SA treated by HCl at different pH values. The molecular weight of SA was 273 kDa. The concentration of SA was 10.0 mg mL^−1^.

The mechanical property of the helix was unclear for SA. In rheological tests, when the pH of the SA dispersion was adjusted from 6.5 to 2, shear-thinning effects of non-Newtonian fluid were observed for all samples (Figure 3a) [37]. Their apparent viscosities (*η*) all exhibited obvious flat areas around 0.3 Pa·s at low shear rates. But the zero shearing viscosity (*η*_0_) was slightly increased from 0.3 to 0.5 Pa·s when the pH was decreased to 4.0. Meanwhile, the flat areas gradually narrowed with decreased pH, indicating that the presence of helix changed the interactions within the SA physical networks. The *η* continued decreasing under high shear rates, indicating pseudoplastic behavior. However, for the shear rate of 148 s^−1^ applied in the wet spinning process, the *η* of the SA dispersion was not significantly changed by the pH values. The viscoelasticity for SAs of different pH values was also studied through dynamic rheological analysis. An angular frequency (*ω*)-dependent increase in storage modulus (*G*′) and loss modulus (*G*″) was observed for SA of different pH values (Figure 3b and Figure S6). Interestingly, *G*′ at low frequency exhibited stepwise increases when the pH was decreased from 6.5 to 2.0, while no obvious change for *G*″ was observed until the pH was lower than 3.0. This evidence implies that the helical confirmation possessed a higher elastic modulus than the random coil, and mainly enhanced elasticity other than viscosity in SA physical networks [38]. *η*, *G*′, and *G*″ overlapped by certain degrees in high-frequency areas, indicating a similar final physical state for SA and acidified SA. In acidified SA dispersions, the ratios of -COOH to -COO^−^ increased with decreased pH values. Growing contents of -COOH were more favorable to the forming of stronger intermolecular interactions, such as hydrogen bonding. Such weak interactions may not only stabilize the helical conformation of SA, but also change the rheological behavior of SA dispersions [39].

### 3.3. Helix Reinforced Alginate Fibers

**Figure 4:**
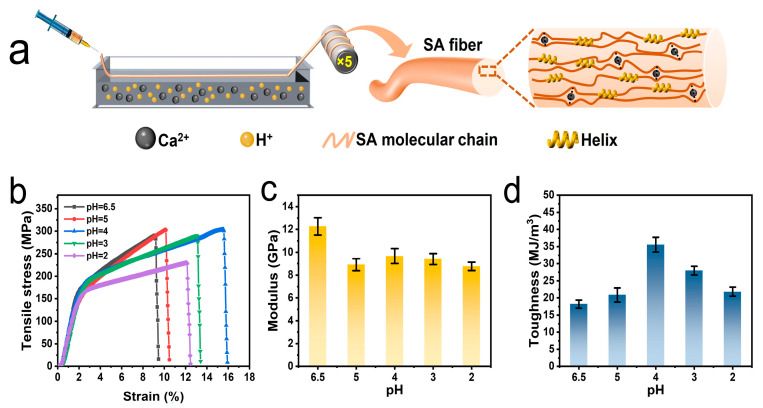
Fabrication and mechanical properties of alginate fibers. (**a**) Schematic illustrations of wet-spinning process. The mechanical properties, including (**b**) stress–strain curves, (**c**) calculated modulus and (**d**) toughness based on stress–strain curves (*N* = 10) are presented for alginate fibers fabricated from HCl/CaCl_2_ at different pH values. The spinning dope was 4 wt. % SA of 273 kDa.

Alginate fibers were fabricated by a typical wet-spinning process. In the HCl/CaCl_2_ coagulation bath, both the fracture tensile strength and strain began to increase with decreased pH values. The fracture tensile strength values were 294.2 ± 8.9 and 308.0 ± 6.8 MPa for the coagulation baths of pH = 5 and 4, respectively (Figure 4b, Table S3). The corresponding strain was 11.8 ± 2.4 and 15.3 ± 0.3%. However, their modulus was in the range of 8.9–9.7 GPa, which is significantly lower than that of SA fibers (Figure 4c). The decreased modulus may result from the inhibited coordination of acidified SA with Ca^2+^. It should be mentioned that a strain-hardening behavior was observed in the stress–strain curves, which existed in other reports of alginate fibers and hydrogels [40,41,42]. This strain-hardening behavior resulted from the enhanced alignment and orientation of sodium alginate chains in the fiber structure. In the stress/strain curves of this work and the reported literature, yielding points of alginate fibers usually emerged at 2–3% of strain. Below the yielding points, the entangled amorphous chains were stretched with gradually improved chain alignment and orientation. But the feature of high ionic-crosslinked density could only provide limited fiber deformation for extension, after which yielding points were reached. After the yielding points were met, higher stress was required to break the aligned alginate chains, exhibiting the strain hardening behavior of alginate fibers.

**Figure 5:**
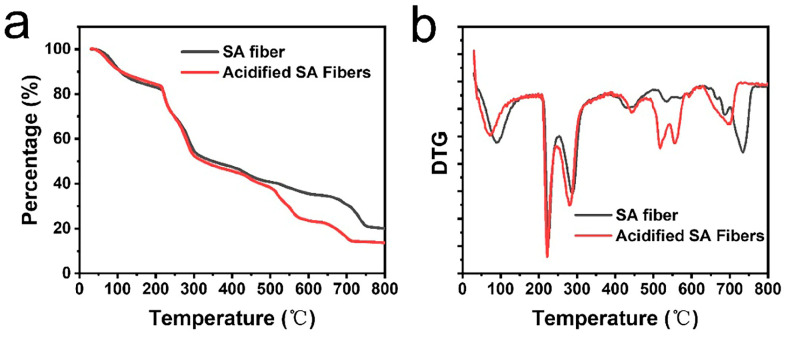
TGA data including (**a**) weight loss percentage and (**b**) and differential thermogravimetric curves (DTG) for SA fibers from a CaCl_2_ bath and acidified SA fibers from an HCl/CaCl_2_ bath of pH 4.

Thermo-gravimetric analysis (TGA) curves show no obvious changes in the weight loss temperature range. The TGA curves could be mainly divided into three regions, namely 30–150, 200–350, and >500 °C. The first stage in the range of 30–150 °C resulted from the evaporation of absorbed water. Due to decreased hydrophilicity, the weight loss percentage of acidified alginate fibers was 1.46% lower than that of SA fibers. The temperature range of 200–350 °C was the main decomposition region for alginate, in which the decarboxylation and thermal degradation of polysaccharides took place [43]. In this region, the acidified alginate fibers lost 3.04% more weight than SA fibers, which resulted from a lower calcium crosslinking degree in acidified alginate fibers. Thus, acidified alginate fibers exhibited decreased thermal stability. The decreased thermal stability became more obvious when the temperature was higher than 500 °C, where alginate underwent carbonization and formed Ca and Na salts. Due to a lower content of calcium, the decomposed residual of acidified alginate fibers was 6.36% lower than that of SA fiber, indicating a decreased Ca^2+^ crosslinking degree (Figure 5). Acidified alginate fibers absorbed water slower than SA fibers in PBS solution. The weight of SA fibers was increased to ~700% after 3 days in PBS, while that of acidified alginate fibers was only ~400% after 4 days in PBS (Figure S5). Nevertheless, due to enhanced strength and elongation, the fiber toughness was increased by 74% to 35.5 ± 2.1 MJ m^−3^ compared with SA fibers (Figure 4d). As for fibers fabricated at pH = 2.0, the acidity was too high to form even structures. Sharp decreases in fracture tensile strength and elongation were observed (Figure 4b). This evidence confirms that in the HCl/CaCl_2_ coagulation bath, the acidity-regulated helix can efficiently improve fiber elongation and toughness without sacrificing the strength, thus reducing the brittleness of alginate fibers.

**Figure 6:**
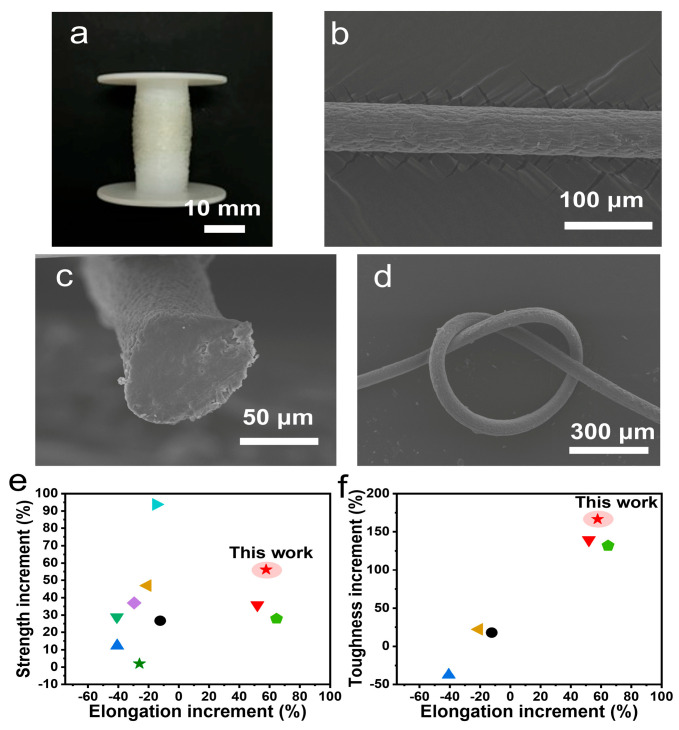
Characterizations of helix-modified alginate fibers. (**a**) A photograph of alginate fibers collected on a roll. SEM images of (**b**) side view of an alginate fiber and (**c**) a cross-section view. (**d**) An SEM image of knotted alginate fiber. (**e**,**f**) Mechanical property comparison of helix-modulated alginate fibers with other reinforced alginate fibers concerning strength (**e**) and toughness (**f**) increment. The additives include 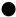 hydroxyapatite [44], 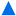 sodium polyacrylate [20], 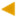 Antarctic krill protein [45], 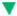 Kapok [46], 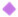 hemp [46], 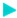 graphene oxide [47], 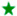 graphene [48], and 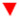 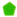 bacteria cellulose nanofibers [54].

The HCl/CaCl_2_ coagulation bath may be suitable for the scale production of reinforced fibers (Figure 6a). The SEM images reveal the one-dimensional fiber morphology, wherein the surface is covered by some shallow folds (Figure 6b). The average diameter was 69.8 ± 5.9 μm. The fiber cross-section view is dense, with no large structural defects but small voids observed (Figure 6c). The fiber flexibility was improved and fibers were easy to knot without brittle fracture (Figure 6d). The mechanical properties of helix-modified alginate fibers were comprehensively compared (Figure 6e,f). For elongation, acidified alginate fibers were superior due to a high content of extendable helix (17.3%) being involved. Further, the helix possessed a higher modulus than random coil. Acidified alginate fibers also possessed enhanced fracture tensile strength, exhibiting the synchronous enhancement of strength, elongation and toughness.

Compared with other reinforced alginate fibers, when stiff reinforcing units such as hydroxyapatite, Antarctic krill protein, kapok, hemp, graphene oxide (GO), and bacterial cellulose were added, their mechanical properties exhibited enhanced strength but negative increments (−54.3~−12.3%) in elongation (Figure 6e, Table S5) [20,44,45,46,47,48,49,50,51,52]. This was mainly contributed by the replacement of extendable moieties with stiff ones, which decreased fiber extendibility and caused the problem of enhancing strength by sacrificing elongation. As for toughness, these stiff reinforcing units resulted in either low increments (14.4~22.3%) in toughness due to greatly enhanced strength, or largely reduced toughness due to severely sacrificed elongation (Figure 6f, Table S5). However, for bacterial cellulose-reinforced alginate fibers, the additional twisted helix was reported to show a similar principle to helical structures, whereby it could enhance strength, elongation and toughness [40]. These result confirm that extendable helical structures were effective to reduce the fiber brittleness. Simultaneously enhanced strength, elongation and toughness have also been termed unusual mechanical properties [53].

## 4. Conclusions

In summary, this work demonstrates a novel method used to reduce the brittleness of alginate fibers. By simply adding HCl into the CaCl_2_ coagulation bath, some of the sodium alginate molecules underwent helical conformational changes. The helical structures contained a 2_1_-helix from G blocks and an antiparallel twofold helix from M blocks. The helix was extendable and possessed higher modulus than the random coil. When alginate coagulated in HCl/CaCl_2_, the helix replaced some amorphous polymer chains, exhibiting improved mechanical performances. Increased fiber elongation from 9.4 to 15.3% and toughness from 20.4 to 35.5 MJ m^−3^ were achieved without sacrificing the fracture tensile strength, reducing fiber brittleness. Unlike conventional ways of modulating mechanical properties by using additives, the unavoidable strength–toughness trade-off is not observed in helix-modulated alginate fibers. Since no obvious changes were made to the wet-spinning process, this work may potentially be useful for industrial exploitation. The improved elongation and toughness will broaden the biomedical applications of alginate fibers.

## Data Availability

The original contributions presented in the study are included in the article/Supplementary Materials, and further inquiries can be directed to the corresponding authors.

## Supplementary Materials

The following supporting information can be downloaded at: https://www.mdpi.com/article/10.3390/ma18112619/s1, Figure S1. The GPC data of SA of 273 kDa. Figure S2. The absorption spectra of acidified SA at different pH values. The concentration of SA was 1.0 mg mL^−1^. Figure S3. Absorption spectra of SA and SA/MB mixture in the range of 200–800 nm. Figure S4. SEM photograph of SA fibers fabricated from coagulation bath of 5 wt. % of CaCl_2_. Figure S5. The mass change percentage of (a) SA fibers and (b) acidified fibers in PBS solution. Figure S6. Rheological measurements of acidity-regulated helix in SA dispersion. Loss modulus (G″) of SA treated by HCl at different pH values. The molecular weight of SA was 273 kDa. The concentration of SA was 10.0 mg mL^−1^. Table S1. A list of the fitted content of secondary structures in HCl-treated SA. Table S2. The CD signal corrected by the relative absorbance of SA/MB at 570 and 665 nm. Table S3. A list of mechanical properties of SA fibers and helix-regulated alginate fibers. Table S4. A table of TGA data for SA fiber and acidified SA fibers. Table S5. A comparison of mechanical properties for alginate fibers and other fibers.
