# Supplementary material for: Structure Characterization and Mechanical Properties of Acidity-Induced Helix of Alginate and Fibers
Source: Materials (Basel). 2025 Jun 3;18(11):2619. doi: 10.3390/ma18112619 (PMC12156795; doi:10.3390/ma18112619)
Supplement: Supplementary file 1 [file materials-18-02619-s001.zip › materials-3634724-supplementary.pdf]

Supporting Information

Structure Characterization and Mechanical Properties of Acidity-Induced Helix of Alginate and Fibers

Jinhong Yang [1,†], Na Sun [2,†], Xuelai Xie [1], Zhangyu Feng [1], Na Liu [1], Kai Wang [1,3,*] and Min Lin [1,*]

[1] State Key Laboratory of Bio-Fibers and Eco-Textiles, College of Materials Science and Engineering, Qingdao University, Qingdao 266071, China; 19863709329@163.com (J.Y.); xxl15550311426@163.com (X.X.); feng101811@163.com (Z.F.); 17363607490@163.com (N.L.)

[2] College of textiles and clothing, Qingdao University, Qingdao 266071, China; sunduanna2009@163.com

[3] Institute of Flexible Electronics (IFE), Northwestern Polytechnical University (NPU), Xi'an 710072, China

* Correspondence: kaiwang@nwpu.edu.cn (K.W.); linmin0401@qdu.edu.cn (M.L.)

† These authors contributed equally to this work.

1. The conversion of breaking tensile strength from unit cN/dtex to unit Pa.

$$strength(Pa) = \frac{F}{S} = \frac{F}{m/\rho L} = \frac{F}{m/L} \bullet \rho \quad \text{(Equation S1)},$$

in which F *is* breaking force (N), m is fibre mass (g), L is fibre length (10000 m) and ρ is mass density of fiber (1.6×10^{6} g m^{-3}).

$$strength(N/dtex) = 100 \times strength(cN/dtex) = \frac{F}{m/L} \quad \text{(Equation S2)}$$

Therefore, for alginate fibers,

$$strength(Pa) = 100 \times strength(cN/dtex) \bullet \rho = 1.6 \times 10^{8}\, strength(cN/dtex) \quad \text{(Equation S3)}$$

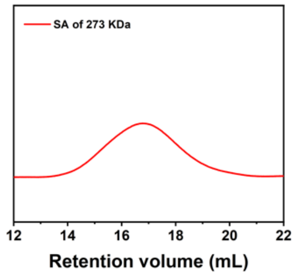

Fig. S1. The GPC data of SA of 273 kDa.

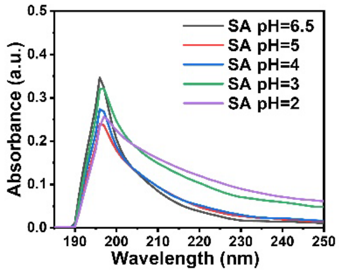

Fig. S2. The absorption spectra of acidified SA at different pH values. The concentration of SA was 1.0 mg mL^{-1}.

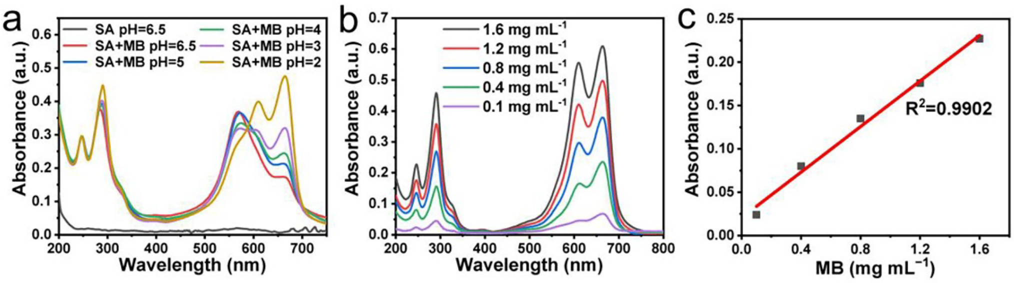

Fig. S3. (a) Absorption spectra of SA and SA/MB mixture in the range of 200-800 nm. (b) Absorption spectra and (c) calibration curves at 247 nm of MB at different concentrations.

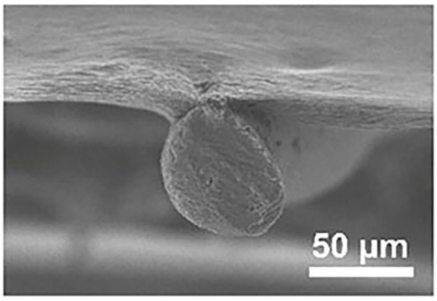

Fig. S4. SEM photograph of SA fibers fabricated from coagulation bath of 5 wt% of $CaCl_2$.

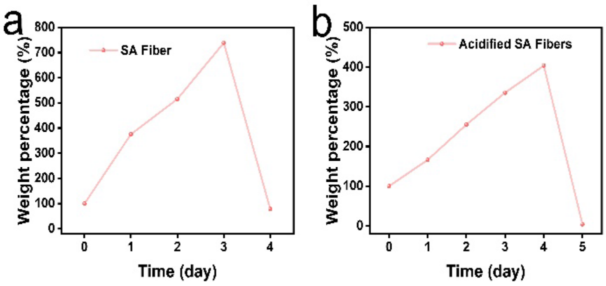

Fig. S5. The mass change percentage of (a) SA fibers and (b) acidified fibers in PBS solution.

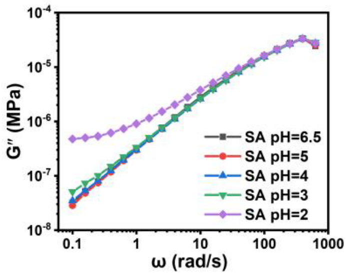

Fig. S6. Rheological measurements of acidity regulated helix in SA dispersion. Loss modulus (G'') of SA treated by HCl at different pH values. The molecular weight of SA was 273 kDa. The concentration of SA was 10.0 mg mL^{-1}.

Table S1. A list for fitted content of secondary structures[a].

Sample	Helix		Antiparallel			Parallel
	Regular	Distorted	Left-twisted	Relaxed	Right-twisted	
pH=6.5	0	6.5±1.3	0	9.1±4.9	12.2±2.3	3.8±0.2
pH=5	1.5±1.7	6.8±2.4	0.8±1.4	1.4±1.2	17±3.9	9.2±1.5
pH=4	6.2±1.4	11.1±1.1	0	0	20.7±4.4	8.2±0.6
pH=3	10.2±3.1	9.8±0.6	0	0	22.2±2.8	5.2±0.9
pH=2	9.6±1.1	10.4±0.7	0	0	22.6±3.5	6.3±0.8

[a] The contents of helix, β-sheet and random coil were roughly estimated through an online tool named BeStSel™ (https://bestsel.elte.hu/index.php). The tool is provided by ELTE Eötvös Loránd University, Budapest, Hungray.

Table S2. The CD signal corrected by the relative absorbance of SA/MB at 570 and 665 nm.

	570 nm		665 nm	
	Relative Abs.	CD (mdeg)	Relative Abs.	CD (mdeg)
SA/MB, pH=6.5	1[b]	-3.90	1[b]	6.99
SA/MB, pH=5	0.886	-29.88	1.104	27.15
SA/MB, pH=4	0.864	-61.44	1.306	43.74
SA/MB, pH=3	0.743	-69.59	1.589	32.64
SA/MB, pH=2	0.940	6.39	2.399	-0.307

[b] The refference absorbance was SA/MB at pH=6.5. The intensity at 570 and 665 nm was seperately set as 1.

Table S3. A list of mechanical properties of SA fibers and helix regulated alginate fibers.

Sample	yield strength	Strain (%)	Stress (MPa)	Modulus (GPa)	Toughness (MJ m^{-3})
pH=6.5	190.4	9.4±0.6	274.4±8.1	12.4±0.6	20.4±0.3
pH=5	177.2	11.8±2.4	294.2±8.9	8.9±0.5	20.9±2.1
pH=4	193.3	15.3±0.3	308.0±6.8	9.7±0.7	35.5±2.1
pH=3	190.3	13.7±0.9	273.9±13.8	9.4±0.5	27.9±1.2
pH=2	172.5	12.3±1.1	217.4±11.6	8.8±0.4	21.8±1.3

Table S4. A table of TGA data for SA fiber and acidified SA fibers.

Sample	SA Fiber	Acidified SA Fibers	**Reaction**
30℃-150℃	14.33%	12.87%	evaporation of absorbed water
200℃-350℃	31.25%	34.29%	decarboxylation and thermal degradation
>500℃	20.02%	13.66%	carbonization and $CaCO_3$

Table S5. A comparison of mechanical properties for alginate fibers and other reinforced biomass fibers.

	Elongation (%)	Strength (MPa)	Toughness (MJ m^{-3})	Reinforced additives	Elongation (%)	Strength (MPa)	Toughness (MJ m^{-3})	References (Supporting information)
This work	9.4	274.4	20.4	$CaCl_2$ of pH 4	15.3	308	35.5	This work
Alginate fibers	23.5	121.4	15.7	Hydroxyapatite	20.6	153.8	18.5	[44]
	11.8	0.57 cN/dtex	5.6	Sodium polyacrylate	7.0	0.64 cN/dtex	3.5	[20]
	12.0	1.8 cN/dtex	21.9	Antarctic krill protein	9.5	2.6 cN/dtex	26.8	[45]
	2.1	135 cN	--	Kapok	1.2	174 cN	--	[46]
	2.1	135 cN	--	Hemp	1.5	185 cN	--	
	16.8	320	--	Graphene oxide	14.3	620	--	[47]
	7.6	199	--	Graphene	5.6	203.8	--	[48]
	35	130	37.5	--	16.0	173	23.0	[54]
	15.6	175	16.0	Bacterial cellulose	7.5	420.4	18.3	[54]
	7.5	420.4	18.3	Helical twisting	10.5-16.2	494-504.2	31.4-44.8	
